# *PLoS Pathogens—*A High-Impact Journal for Pathogen Research

**DOI:** 10.1371/journal.ppat.0010012

**Published:** 2005-09-30

**Authors:** John A T Young

On behalf of the editorial board and the PLoS staff, I welcome you to the inaugural issue of *PLoS Pathogens*. We hope that you enjoy reading the exciting articles in this first issue. These papers were selected because each represents a breakthrough in understanding the biology of pathogens and pathogen–host interactions. For example, in a highly innovative study (DOI: 10.1371/journal.ppat.0010007), Orr and colleagues have taken advantage of recombinant herpes viruses, using the mouse model system to demonstrate the importance of major histocompatibility complex (MHC) Class I–driven antigen cross presentation for the induction of virus-specific CD8+ T cells. They show that viral inhibition of MHC class I function is important for viral entry, replication, and survival in the central nervous system. Also, Wang et al. (DOI: 10.1371/journal.ppat.0010009) elegantly describe a critical role for a specific region of the *Plasmodium* circumsporozoite protein in sporozoite exit from oocysts, a process that might represent a new therapeutic target for treating malaria. These are precisely the type of high-impact research articles that we envisage appearing in *PLoS Pathogens*.

From the beginning, the editorial board committed to publishing groundbreaking findings on bacterial, fungal, parasitic, prionic, and viral pathogens. By doing so, we intend to showcase the most important new ideas and results, particularly those of broad interdisciplinary interest. Our goal, achieved in this inaugural issue, is to bring to you articles detailing significant and substantive scientific advances that merit your attention and that of the broad readership of *PLoS Pathogens*.

The readership of this journal is, indeed, as varied as it is vast. As an open-access journal, *PLoS Pathogens* makes new findings that can shape and guide research efforts and scientific progress immediately and freely available to the broadest possible global audience. The free availability of key, new information and findings encourages the active exchange of ideas within and throughout the pathogen research community worldwide. The addition of a short, nontechnical summary accompanying each article in *PLoS Pathogens* adds another dimension to our accessibility. These synopses represent an important mechanism for sharing scientific advances with nonspecialists, and facilitating communication between scientists and members of the general public.

Research articles are the primary focus of *PLoS Pathogens* because the editorial board has recognized the critical need for a high-impact journal in our field. Consequently, we have set a very high bar for manuscript acceptance. Our review process is extremely rigorous, but timely, and is receiving high marks from our authors. Indeed, many have told us that they find the detailed and constructive feedback they receive to be extremely useful.

In addition to research articles, *PLoS Pathogens'* readers will find Opinions and Reviews in most monthly issues. Opinions Editor Marianne Manchester outlines her vision for this section in an accompanying article in this issue; I encourage you to read and respond to her ideas and plans. Brett Finlay, *PLoS Pathogens'* Reviews Editor, has solicited a number of high-quality, cutting-edge Reviews, and is also capitalizing on the great expertise of the editorial board to invigorate and develop this section of the journal. But he is also keen to hear your suggestions and ideas about important, new topics that would capture your and your colleagues' interest.

*PLoS Pathogens* is launching at an important time, when the pathogen research community has much to learn from each other and to share with the global community. We invite you to be a part of it—as readers, learners, authors, or reviewers—your contributions will enrich us all. Send us your presubmission inquiries and manuscript submissions at http://plospathogens.org—your best ideas and your best work! 

